# Spousal Loss and Cognitive Functioning: Do Pre-Loss Marital Quality and Gender Matter?

**DOI:** 10.1093/geroni/igaa057.1178

**Published:** 2020-12-16

**Authors:** Joohong Min, Jieun Song

**Affiliations:** 1 Jeju National University, Jeju, Cheju-do, Republic of Korea; 2 Waisman center, Madison, Wisconsin, United States

## Abstract

Prior research has found that the risk of cognitive decline increases after the death of a spouse. In general, the impact of life transitions is contingent on contextual factors such as socio-demographic characteristics or relationship quality. However, there is limited research on how marital quality before spousal loss and gender influence the association between spousal loss and cognitive change. The current study examines the effects of spousal loss on change in cognitive functioning as well as the moderating effects of pre-loss marital quality and gender. Data from two waves of the Midlife in the United States (MIDUS) study were analyzed (MIDUS2: 2004-05, MIDUS3: 2013-14). The analytic sample consists of two groups: (1) 179 bereaved adults who were age 54 or older at MIDUS2 (M = 65.2, SD = 9.5) and whose spouses died between MIDUS2 and MIDUS3, and (2) 179 non-bereaved adults, matched with the bereaved group on age and gender, who did not experience spousal loss between the two waves. Cognitive function was assessed via BTACT (Brief Telephone Adult Cognition Test) at both waves. Regression results show that both pre-loss marital quality and gender significantly moderate the association between spousal loss and change in cognitive functioning. Specifically, relative to their counterparts, men and those who reported better marital relationships prior to spousal death had a greater risk of cognitive decline after a spouse’s death. The findings suggest the significance of pre-loss marital quality and gender for cognitive changes in widowhood and have implications for the development of efficient interventions

